# Current state of the art in the transmission of mitochondrial DNA pathogenic variants: reviewing preventive-therapeutic alternatives

**DOI:** 10.1007/s10815-026-03888-0

**Published:** 2026-05-05

**Authors:** Marta Reguera-Cabezas, Felipe Martínez-Pastor, Cristina Soriano-Úbeda

**Affiliations:** 1 Assisted Reproduction Unit, Marqués de Valdecilla University Hospital, Santander, Spain; 2 Department of Molecular Biology (Cell Biology), University of León, León, Spain; 3 Institute of Animal Health and Livestock Developemnt (INDEGSAL), University of León, León, Spain; 4 Department of Veterinary Medicine, Surgery, and Anatomy, University of León, León, Spain

**Keywords:** Assisted reproduction, Genetics, Heteroplasmy, Mitochondrial diseases, Mitochondrial replacement

## Abstract

This review analyzes strategies to prevent or reduce the transmission of diseases caused by pathogenic variants in mitochondrial DNA (mtDNA). Among these, we will focus on prenatal screening, preimplantation genetic testing, gene-editing techniques, other molecular tools, and selected heterologous mitochondrial replacement techniques (MRTs), explaining their status and the uncertainties surrounding their clinical application. After this analysis and review, we recognise the limitations of the efficacy of prenatal and preimplantation genetic testing for mitochondrial DNA pathogenic variants, the legal constraints on gene editing, and the status of mitochondrial replacement techniques. MRTs are a safe and possibly more effective alternative for preventing diseases caused by mitochondrial DNA pathogenic variants.

## Introduction

The mitochondrial genome was sequenced more than four decades ago [1]. Since then, research into the role of mitochondrial dysfunction in human pathologies has advanced considerably [2, 3]. Mitochondrial diseases are a very heterogeneous group of genetic diseases caused by failures in the oxidative phosphorylation system (OXPHOS) with different inheritance mechanisms: autosomal and X-linked, in a Mendelian pattern for variants in nuclear DNA (nDNA), and mitochondrial, maternal in a vertical non-Mendelian pattern for variants in mitochondrial DNA (mtDNA) [4]. Maternal inheritance of mtDNA is a passive consequence of eliminating the paternal mtDNA, most likely during spermatogenesis [4]. Thus, mtDNA-derived mitochondrial dysfunction is exclusively of maternal origin [5, 6].

The mtDNA replicates during the cell cycle, and pathogenic mtDNA variants are randomly distributed between daughter cells during cell division [7, 8]. Most commonly, cells harbor mixed populations of mtDNA, a phenomenon termed heteroplasmy [9, 10]. Conversely, homoplasmy occurs when a cell’s mitochondrial content contains a single mtDNA variant.

In oocytes, mtDNA replication is initiated in migrating primordial germ cells, and mtDNA variants are segregated during mitotic divisions of germ cell precursors [11]. The mechanism of mitochondrial distribution during germ cell development, the so-called “bottleneck,” can lead to a random change in the pathogenic mtDNA load [10, 12]. This process presumably involves two distinct phases: a first one characterized by a sharp reduction in mtDNA copy number before primordial germ cell specification, followed by a progressive increase in mtDNA copy number as the oocyte maturation progresses until it achieves the metaphase II stage, in which the mtDNA copies are maximum and maintained up to fertilization; and a second phase starting at the pronuclear stage of the zygote, where the mtDNA copy number is progressively reduced during the succession of the preimplantation embryo development stages [5, 13, 14]. Additionally, mtDNA can segregate in somatic tissues [15].

The pathologies caused by mtDNA variants are maternally transmitted and result in rare, incurable, and potentially prematurely fatal diseases with progressive deterioration of the patient [16], causing a wide range of pathophysiological manifestations. Among them, highlighted for their clinical severity, we find Leigh syndrome (LS) [17], Leber hereditary optic neuropathy (LHON) [18], mitochondrial encephalomyopathy with lactic acidosis (MELAS) [19], myoclonic epilepsy of irregular red fibres (MERRF) [20], and Leigh syndrome with degenerative neurological condition associated with ataxia and retinitis pigmentosa (NARP) [21]. A single pathogenic variant causes some of these diseases, whereas others can arise from different mutations in the same gene [22, 23]. There are also other types of alterations caused by single mtDNA deletions or duplications, such as sporadic progressive external ophthalmoplegia (PEO) [24], Kearns-Sayre syndrome (KSS) [25], and Pearson syndrome [26]. The aetiology of these diseases affects OXPHOS, leading to symptoms in vital organs with high energy requirements, such as the brain, heart, liver, and kidneys [2]. The severity of these pathologies is highly variable and difficult to predict, and depends on the type of variant and the degree of heteroplasmy [27–29]. Nevertheless, the mtDNA variant load is not always indicative of whether a carrier will become symptomatic or of disease severity, although heteroplasmy thresholds have been proposed in some cases [30]. Thus, the dynamics of heteroplasmy are among the most clinically challenging aspects of mtDNA diseases and pose a challenge to preventing transmission [31–33].

In countries where advanced technologies of cell editing are not allowed, women carrying pathogenic mtDNA variants must give up having a genetic link to their children if they wish to avoid transmitting the disease completely. Their only lawful options are adoption or in vitro fertilization with donated oocytes [34]. However, the risk can be reduced by using preimplantation genetic testing for monogenic disorders (PGT-M) or prenatal diagnosis (PND), and by assessing whether heteroplasmy levels are acceptable [35–37]. Nonetheless, to date, the only plausible strategy to actively reduce the risk of pathogenic variant mtDNA transmission is the so-called Mitochondrial Replacement Techniques (MRTs), which involves replacing the cytoplasm of the affected oocyte with that of a healthy donor [34, 38, 39]. Moreover, PGT-M and PND are not helpful when a woman is diagnosed with mitochondrial disease with homoplasmy or severe heteroplasmy, as the likelihood of producing oocytes with low heteroplasmy is low.

MRTs are, by definition, forms of heterologous cytoplasmic replacement. Hailed as one of the most significant recent advances in reproductive medicine [40], MRTs currently represent the only effective approach for reducing the risk of transmitting pathogenic mtDNA variants in cases of homoplasmy, while preserving a maternal genetic connection [41] (of course, also in cases of heteroplasmy). However, MRTs still risk transferring a small portion of the patient's cytoplasm — containing pathogenic mtDNA variants — into the healthy oocyte or zygote. This is due to the high concentration of mitochondria in the perinuclear region, which remains associated with the maternal nucleus during the procedure. Although the resulting mtDNA carryover is typically estimated to be less than 1–2%, it may reach levels that lead to clinically significant and potentially unacceptable levels of heteroplasmy [42–45].

In this review, we first consider the different approaches to testing for mitochondrial diseases and to preventing their inheritance or reducing their impact, including experimental approaches. Subsequently, we examine current developments in MRTs, assessing the various approaches and their strengths and limitations. As noted previously, the use of MRTs is prohibited in many countries. Only the United Kingdom [46] and Australia [47] have regulated MRTs for therapeutic purposes; however, MRTs are currently authorized and practiced only in the former [48], yet several births resulting from these techniques have occurred in several countries without a legal framework to regulate them [40, 49]. The legal development and the discourse on the ethical and technical dimensions are outside the scope of the present review and have been discussed elsewhere [50, 51].

## Current screening and preventive approaches to mitochondrial diseases

Advances in understanding and in the development of screening tools have significantly enhanced the prevention of inherited genetic disorders. Nonetheless, depending on the specific pathology, implementing these screening techniques in clinical practice may be challenging. In situations where mitochondrial disease is suspected, preconception genetic counselling aims to perform a detailed assessment to identify the genetic variant as precisely as possible. This initial diagnosis, which focuses on detecting pathogenic mtDNA variants in the woman, is crucial for obtaining reliable information on the specific variants present and their heteroplasmy levels. It serves as the foundation for preventive strategies and informs the counselling process regarding disease risk, prognosis, available treatments, and measures to reduce the likelihood of transmission [52, 53].

Consequently, genetic counselling offers tailored preventive approaches that consider the medical, psychological, and cultural contexts of the individual or family undergoing genetic evaluation. It also empowers women or couples to make informed reproductive decisions [54]. After molecular testing, the patient and their family should refer to a specialized service for genetic counseling and family follow-up, recognizing that the risk of recurrence and for other family members depends on whether the pathology arises from a nuclear-encoded gene or an mtDNA variant [55]. In this regard, the European Neuro Muscular Centre (ENMC) has published guidelines on reproductive options in mitochondrial disease that may be of interest to those who wish to explore this further [56].

This section outlines the available screening techniques for genetic diseases, including preimplantation and prenatal testing, which are currently the only options in countries specifically forbidding MRTs.

### Preimplantation genetic testing for monogenic disorders and the specific case for mtDNA pathogenic variants

PGT-M is a preventive screening method performed on embryos generated through in vitro fertilization. In the case of mitochondrial diseases caused by mutations in nuclear genes, PGT-M would be the screening and preventive technique of choice [3, 12, 30, 57]. In 2005, the first successful application of PGT for NARP was published; however, the authors had already highlighted discrepancies between the segregation patterns of different mtDNA variants and the resulting limitations in the use of PGT [58]. In the case of preimplantation genetic testing for mtDNA pathogenic variants (PGT-MT), the objective of the screening is to select embryos with low mutant load from women with moderate levels of heteroplasmy [12, 55, 59], with the aim that the fetal load is below a critical threshold [53]; however, in women with severe heteroplasmy or homoplasmy, PGT-MT is of little or no use, since all resulting embryos would carry an unacceptably high mutant load [60]. This situation significantly limits its application and does not guarantee the birth of disease-free offspring [61, 62]. Moreover, the possibility of selecting embryos with minimal heteroplasmy is influenced by individual patient factors, not only the degree of heteroplasmy but also the specific mtDNA variation, age, ovarian reserve, and other yet unidentified factors [60–62]. Specifically, the extent of the bottleneck, and possibly the critical threshold of mutant load for embryonic/foetal survival, varies across different pathogenic mtDNA mutations, which complicates genetic counselling and pre-implantation screening [63]. However, some common pathogenic variants are more likely to exhibit a skewed distribution in the resulting embryos, increasing the likelihood of identifying embryos with low load even when the mother has a relatively high heteroplasmy level [37, 39].

We have to consider not only the uncertainty of embryo selection, but also other factors out of the scope of the present revision: the concomitant risks of blastocyst biopsy [64], the validity of the sampled cells as representative for the whole embryo, and the potential for heteroplasmy levels to change during the pregnancy [53, 65]. Moreover, a comprehensive clinical assessment of mitochondrial disease may include the collection of biological samples from multiple sources (e.g., blood, skin, muscle) from the mother and relatives, to fully understand heteroplasmy levels and their clinical significance [39].

After PGT-MT, and if pathogenic variant-free embryos are not available, the ART centres must consider which embryos are below a predetermined threshold of pathogenic variant mtDNA, offering specialized advice to patients who would have to make decisions about accepting or rejecting the use of heteroplasmic embryos for transfer to the uterus. The lower the pathogenic mtDNA load in the embryo, the lower the risk of disease manifestation in the offspring [66–68]. Nevertheless, depending on the regulations in individual countries and professionals’ opinions, the outcomes of embryo transfer following PGT-MT may vary [56, 69, 70]. This heterogeneity, from conservative and stringent transfer thresholds to more liberal transfer policies, highlights the problematic balance between maximizing the chance of having an unaffected child and the highest chance of achieving pregnancy with minimal risk of any children born developing mtDNA disease. A recent report [53] presented 39 cases of PGT-MT, resulting in 18 live births, with heteroplasmy levels in 10 babies ranging from 7% to undetectable.

### Prenatal diagnosis

PND encompasses all interventions to detect genetic diseases in the fetus during gestation. Invasive PND enables the collection of fetal DNA for analysis of genetic diseases and other congenital abnormalities [71]. The most common invasive PND is the chorionic villus sampling (CVS), cordocentesis, and amniocentesis. These were the first techniques employed for PND of Mendelian genetic disorders [72], thereby enabling the detection of mutations in nuclear genes that result in mitochondrial diseases. PND for detecting mtDNA pathogenic variants (PND-MT) is one of the few widely accessible options available to patients at risk of transmitting mtDNA disease [73].

However, PND-MT exhibits numerous weaknesses, some of which are shared with PGT-MT. For instance, chorionic villus sampling can be performed early in pregnancy (by week 10), but the drawback is that pathogenic mtDNA load in the placenta is heterogeneous [74]. In contrast, amniotic fluid analysis is more indicative of the fetus’s status but is typically performed later in pregnancy (around week 16) [73]. Moreover, we must keep in mind that, depending on the tissue, mitochondrial segregation may be different [75] and diverse variants show different pathogenic threshold values for heteroplasmy [3]; nevertheless, many studies have shown that the pathogenic variant load in a prenatal sample can be a reliable predictor for most tissues at birth [55, 73]. Additionally, several authors have proposed models that could reliably predict the outcomes for specific thresholds [54, 76]. In any case, PND is generally not appropriate for homoplasmy [55].

## Other technologies under development

Efforts to target pathogenic mtDNA variants have explored a range of genetic engineering strategies, many of which remain at the experimental stage in non-human and in vitro models. Some of these approaches aim to directly edit mitochondrial genes to correct pathogenic variants, with base editors standing out as promising options [77–79]. These are molecular tools for modifying the base sequence of a DNA fragment, with CRISPR-Cas9 and its variants as the best-known ones [80]. Nevertheless, technical challenges remain, particularly in targeting the guide RNA to mitochondria [81, 82], suggesting that other approaches could be more successful. DddAtox-based or nickase-based mitochondrial base editors show efficiency in different models for editing mtDNA [83], with some studies reporting success in mammal embryos [84, 85].

Other approaches focus on altering heteroplasmy levels by selectively damaging pathogenic mtDNA and promoting the replication of non-pathogenic mtDNA, helping to restore mitochondrial function [7, 77, 86]; for instance, using restriction enzymes [87–89], peptide nucleic acids (PNAs) or short RNA molecules for inhibiting the replication of mutant mtDNA, or mitochondria-targeted designer nucleases (zinc finger nucleases — ZFNs — or transcription activator-like effector nucleases — TALENs) [10, 90, 91].

In addition to the possible ethical debate over whether this kind of editing could be considered heritable modifications of the human genome, a significant challenge of these techniques is the continued occurrence of off-target modifications, including nuclear DNA (nDNA). Despite continuous improvements in systems [92], there remains a long way to go before these technologies are used clinically.

## Mitochondrial replacement techniques: types and status

Given the limitations of PGT-MT and PND in detecting and preventing the transmission of mtDNA pathogenic variants, many researchers have advocated Mitochondrial Replacement Techniques to replace a woman’s abnormal mtDNA variants with a donor’s healthy ones [93, 94]. In any case, they are not curative techniques for the already present mitochondrial disease, but preventive [95].

Basically, MRTs involve transferring the nDNA from a maternal oocyte or embryos to the donor’s oocyte. From the earliest stages of their development, MRTs require an in vitro fertilization (IVF) or intracytoplasmic sperm injection (ICSI) as part of the process (with spermatozoa from a partner or donor), either if the nDNA is transferred to an enucleated mature oocyte before fertilization or if the transference of nDNA is carried out to an enucleated zygote [96–98]. In any case, the involvement of a donor oocyte (with healthy mtDNA) is indispensable. There are currently several technological approaches, some of them legally regulated in a few countries [96]: pronuclear transfer (PNT), maternal spindle transfer (MST), polar body transfer (PBT), and germinal vesicle transfer (GVT) [99].

As mentioned, MRTs are still limited by maternal mitochondrial carryover from the cytoplasm surrounding the nuclear structure, which is inevitably transferred together with the nDNA [100, 101] and varies in proportion depending on the type of MRT [16, 101–104]. Although the proportion of carryover is generally below 2%, it could exceed the threshold for disease manifestation if mitochondrial replication bearing the pathogenic mtDNA variant is predominant [33, 105].

Although other applications are not the aim of the present revision, we must consider that MRTs have been proposed for cytoplasmic oocyte rejuvenation. MRTs have been applied, with live births, to oocytes of women with low ovarian reserve, low oocyte quality, and even idiopathic infertility, for improving embryo development and increasing the chances of a successful pregnancy [106–109].

### Pronuclear transfer

Pronuclear transfer (PNT) requires the formation of two embryos by IVF, one from the maternal oocyte (carrying the pathogenic mtDNA variant) and the other from the donated oocyte (with non-pathogenic mtDNA). Once fertilized and at the zygote stage, the pronuclei are extracted, and the pronuclei from the maternal zygote are transferred to the cytoplasm of the zygote from the donor oocyte, generating a reconstituted embryo [61]. This technique has resulted in live births [110], but to date, the method has been legally regulated only in the United Kingdom [46] and Australia [47].

PNT has been developed over the past decades using various models. As early as the 1980 s, McGrath & Solter, in an effort to study early embryogenesis, demonstrated its potential to produce live offspring in mice [111]. It was not until 2005 that PNT was tested in non-human models to prevent transmission of mtDNA pathogenic variants [112].

PNT shows a significant disadvantage relative to maternal mtDNA carryover, given the high mitochondrial concentration near the pronuclei and transferred to the new zygote. However, when performed carefully, the technique was reported to result in an average carryover of less than 2% [61]. The method was refined in a later study, thereby reducing the risk of pathogenic mtDNA carryover [102], with 8 live births reported [53].

### Maternal spindle transfer

Maternal spindle transfer (MST) requires the transfer of the metaphase plate of the second meiotic division from oocytes with pathogenic mtDNA variants (previously fertilized) into a donor oocyte with healthy mtDNA, which has been previously enucleated [113]. The MST-reconstituted oocyte undergoes ICSI and subsequent in vitro embryo development until transfer to the maternal uterus. MST was technically first described in bovine embryos [114]. Pregnancy to term after MST was first reported in 2001 in mice [115] and in primates in 2009 [43]. The use of MST in non-human models is well supported by research [43, 116], resulting in healthy offspring in a monkey model [117]. This technology was successfully implemented over the last decade, enabling the birth of a few healthy children in countries where MRTs are allowed or unregulated, such as Mexico [34] and Greece [107, 118] (MST in Greece was used to enhance embryo development rather than to remove pathogenic mtDNA).

MST is less invasive and achieves lower residual mitochondrial carryover rates than PNT (e.g., 1% [113]). However, it has other limitations, such as a high sensitivity to micromanipulation because the nuclear envelope, which protects the chromosomes on the metaphase plate, is absent [104]. Therefore, the manipulation of oocytes must be carried out with utmost care to avoid loss of genetic material during spindle extraction, and to minimize the extraction and entrainment of maternal cytoplasm, as it may contain pathogenic mtDNA-bearing mitochondria [119].

### Polar body transfer

Polar bodies harbour membrane-bound genetic material left over from reductive divisions, either in the form of bivalents or chromatids, which are excluded from the oocyte cytoplasm and remain in the perivitelline space of the oocyte or embryo and degenerate at later stages. Considering that polar bodies carry minimal cytoplasm, the first or second polar body from an oocyte with pathogenic mtDNA can be transferred to an enucleated donor oocyte with healthy mtDNA as a form of MRTs [44, 120]. Indeed, Polar Body Transfer (PBT) are the easier MRTs to perform, since the polar bodies are conveniently located below the zona pelucida and easy to retrieve and transfer to the enucleated oocyte.

PBT with the first polar body consists of enucleation of the donor oocyte, transference of the first polar body to the enucleated cytoplasm, and ICSI to fertilize the donor oocyte [121, 122]. Using the second polar body is more complex, since it is necessary to fertilize both oocytes, maternal and donor, with spermatozoa of the same origin. Subsequently, the female pronucleus is removed from the donated zygote and replaced by the second polar body from the maternal zygote [110].

Both first and second PBTs have produced offspring in non-human models with reduced levels of mitochondrial carryover [123], almost undetectable with the first polar body (highly efficient, with only 0.38% carryover) and up to 2% with the second polar body. Nevertheless, the reconstituted embryos were not transferred, and there are no reported pregnancies to date using these techniques [122, 124, 125], despite the successful production of viable offspring in mice in the 90 s [126, 127]. Recently, new developments using mouse and human oocytes have enabled improved transplantation of the second polar body [128].

### Germinal vesicle transfer

Germinal vesicle transfer (GVT) involves transplanting the germinal vesicle—the nucleus of an immature (primary) oocyte—from a patient into an enucleated donor oocyte at the same developmental stage [129, 130]. Compared to other MRTs, GVT is considered less invasive because it manipulates oocytes at the germinal vesicle stage, when the genetic material is still enclosed within the nuclear membrane, providing a degree of protection during the procedure. However, because GVT uses immature oocytes, the reconstructed oocyte must undergo in vitro maturation (IVM), unlike in other MRTs. This additional step is critical to complete meiosis before fertilization and embryo transfer. Unfortunately, current IVM techniques significantly reduce the efficiency of embryo production, representing a significant limitation of the procedure.

To date, only two studies have assessed the outcomes of GVT, both using murine models [129, 130], with low success rates and undetermined mitochondrial carryover. In particular, [130] reported that none of the embryos derived from GVT-reconstructed oocytes developed to the blastocyst stage, primarily due to the suboptimal performance of current IVM protocols [43]. Given these limitations, the clinical use of GVT in humans has not yet been implemented. Significant progress in IVM optimization and a better understanding of mtDNA dynamics are necessary before GVT can be considered a viable option.

### Other technical aspects of MRT implementation

Certain limitations may restrict the use of MRTs in specific situations. A particular case is that of women with low ovarian reserve or a high risk of residual mitochondrial carryover (homoplasmy or a high level of heteroplasmy for the pathogenic mtDNA) [53, 109, 131, 132]. The consensus is to choose the most efficient MRTs in terms of the number of viable blastocysts generated, even if technically challenging. This is obvious when the pool of maternal oocytes is limited and therefore the number of trials; in the case of high risk of pathogenic mitochondrial carryover, the chosen technique must be able to yield a high number of blastocysts per trial, therefore increasing the likelihood of finding at least one with a low carryover rate. Nevertheless, as for other medical therapies, many authors’ opinion is that MRTs should not be recommended for high risk of failure [100, 133, 134].

We must consider that technical constraints can reduce the efficiency of some MTRs. For instance, MST and PNT require the use of cytoskeleton inhibitors, which reduces the success rate [44, 122].

Another potentially relevant (and controversial) factor is the possibility that the mtDNA variant may influence the MRTs efficiency by modulating the cell cycle. Since mitochondrial function is closely linked to cellular energy production and redox balance, factors that affect the biogenesis and differential replication of one mitochondrial variant over another may influence cell-cycle progression [6, 135]. Therefore, specific pathogenic variants in mtDNA could affect the technique's efficacy by altering ATP availability and the intracellular signalling pathways involved in cell-cycle regulation. This hypothesis is currently being explored and requires further experimental validation [135–137].

Despite achieving very low carryover levels in many studies, reversion to maternal mtDNA heteroplasmy has been observed [107], possibly due to unwanted mito-nuclear interactions [138]. To minimize this risk, some authors recommend that the mitochondrial haplogroup match the patient’s [139]; however, others argue that the benefits would not outweigh the resulting reduction in available (compatible) oocytes [140]. To this end, the mitochondrial genome must be sequenced before mitochondrial donation to enable detection of mitochondrial variants in the donor [133]. The use of the same haplotype could minimize tissue segregation effects and reduce the likelihood that one mitochondrial haplogroup is favoured over another during replication [15, 102, 125, 141]. Ultimately, combining MRTs with molecular mtDNA-editing strategies could reduce the risk of phenotypic reversion. Thus, mtDNA editing could enhance the efficacy of MRTs by correcting residual pathogenic mtDNA [101]. Inducing selective mitophagy is another proposed strategy [32]. However, this research remains in its early stages.

Regarding embryonic development to the blastocyst stage, MST and PNT have shown comparable outcomes [61, 118, 142]. However, to ensure that the resulting embryos are suitable, PGT-MT should be performed on these blastocysts to assess the extent of residual mitochondrial carryover, determine the level of heteroplasmy (if present), and detect chromosomal aneuploidies [100]. However, it is essential to note that not all countries permit the use of such screening technologies, and this procedure carries some risk of affecting embryo viability.

Another crucial consideration is the offspring’s long-term well-being. MST, PNT, and PGT-MT are invasive procedures that may impact cellular and nDNA integrity. Therefore, long-term follow-up from birth through adulthood is essential to evaluate the safety of these clinical interventions [100, 143–145].

## Conclusions

MRTs have emerged as a viable experimental option for women with mitochondrial diseases wishing to reduce the risk of transmitting the pathogenic variant to their offspring, particularly when predictive approaches such as PGT-MT or PND do not provide sufficient assurance. The strategies discussed in this context remain experimental and invasive, requiring robust scientific and clinical evidence —including long-term follow-up of the resulting offspring— to confirm their safety and guide their regulated application. Several clinical trials involving MRTs are currently underway, and their outcomes will be critical for determining whether these techniques can be standardized for clinical use. Nevertheless, the legal framework in most countries is still unfavorable.

## Data Availability

No datasets were generated or analysed during the current study.
